# Layer-specific sensory processing impairment in the primary somatosensory cortex after motor cortex infarction

**DOI:** 10.1038/s41598-020-60662-7

**Published:** 2020-02-28

**Authors:** Atsushi Fukui, Hironobu Osaki, Yoshifumi Ueta, Kenta Kobayashi, Yoshihiro Muragaki, Takakazu Kawamata, Mariko Miyata

**Affiliations:** 1 Faculty of Advanced Techno-Surgery, Institute of Advanced Biomedical Engineering and Science, Tokyo Women’s Medical University, Tokyo, Japan; 2 Department of Neurosurgery, Tokyo Women’s Medical University, Tokyo, Japan; 3 Division of Neurophysiology, Department of Physiology, School of Medicine, Tokyo Women’s Medical University, Tokyo, Japan; 4 Section of Viral Vector Development, National Institute for Physiological Sciences, Okazaki, Japan; 5 SOKENDAI (The Graduate University for Advanced Studies), Hayama, Japan

**Keywords:** Sensorimotor processing, Neurophysiology, Stroke

## Abstract

Primary motor cortex (M1) infarctions sometimes cause sensory impairment. Because sensory signals play a vital role in motor control, sensory impairment compromises the recovery and rehabilitation of motor disability. However, the neural mechanism of the sensory impairment is poorly understood. We show that sensory processing in mouse primary somatosensory cortex (S1) was impaired in the acute phase of M1 infarctions and recovered in a layer-specific manner in the subacute phase. This layer-dependent recovery process and the anatomical connection pattern from M1 to S1 suggested that functional connectivity from M1 to S1 plays a key role in the sensory processing impairment. A simulation study demonstrated that the loss of inhibition from M1 to S1 in the acute phase of M1 infarctions could impair sensory processing in S1, and compensation for the inhibition could recover the temporal coding. Consistently, the optogenetic activation of M1 suppressed the sustained response in S1. Taken together, we revealed how focal stroke in M1 alters the cortical network activity of sensory processing, in which inhibitory input from M1 to S1 may be involved.

## Introduction

Sensory information strongly influences motor coordination^1^. Consistently, it is also essential for the restoration of motor performance after stroke and is frequently used for effective neurorehabilitation^2,3^. In primates, both motor dysfunction and somatosensory impairment occur in the acute phase of primary motor cortex (M1) infarction^4,5^. Because rehabilitative therapies are most beneficial when initiated in the acute phase of stroke^6^, understanding how sensory processing is modified after M1 infarction, especially shortly after the injury, is crucial for effective therapies^7,8^

In rodents, the primary sensory cortex (S1) forms reciprocal connections with M1^9–17^. Axons from S1 preferentially innervate layer 2/3 (L2/3) and L5a in M1^10,17^. On the other hand, axons from M1 innervate L1 and L5b in S1 densely^17,18^. Furthermore, both excitatory and inhibitory inputs from M1 increase the acuity of sensory processing in S1^15,18–20^. However, despite many studies on the neural mechanism, including the reorganisation and recovery process of motor dysfunction after M1 infarction^7,8^, the fundamental spatiotemporal dynamics of sensory processing in S1 after M1 infarctions remains unknown.

To address this issue, we used the photothrombotic method on mouse vibrissa M1 (vM1) as a model of focal ischemic stroke and investigated the effect on somatosensory processing in mouse vibrissa S1 (vS1). We found the response reliability to whisker stimulation from each vS1 layer was impaired in the acute phases of vM1 infarctions and recovered in the subacute phase in layer-specific pattern. Following additional simulation and the optogenetic experiments, we propose that the loss of inhibition from vM1 impairs the response reliability in vS1 after vM1 infarction.

## Results

### Laminar patterns of projections from vM1 to vS1

**Figure 1:**
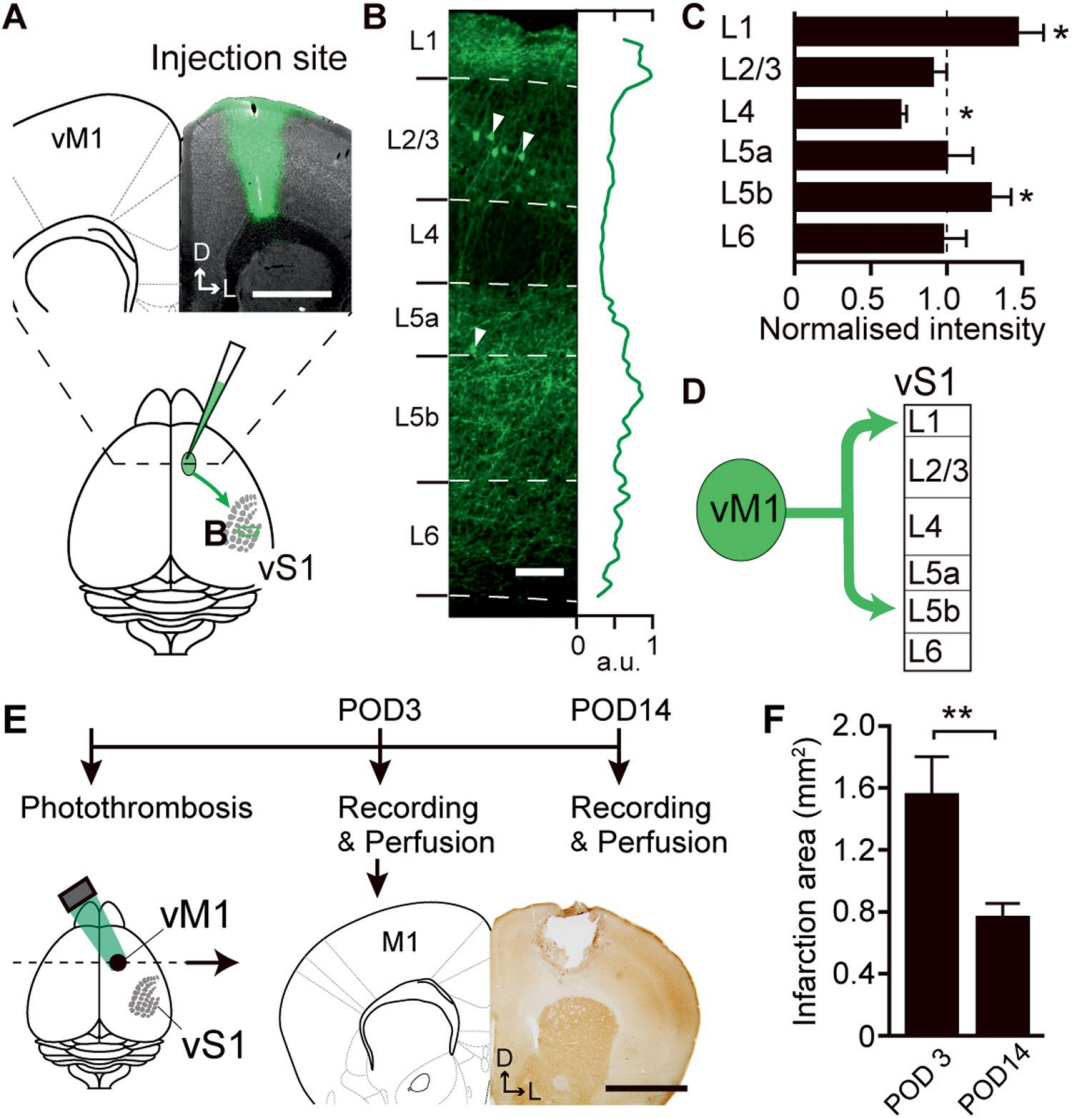
Laminar patterns of vM1 axonal innervations to vS1 and experimental timeline of the vM1 photothrombotic infarction model. (**A**), Anterograde tracer injection into the vibrissa primary motor cortex (vM1). Scale bar, 1 mm. D, dorsal; L, lateral. (**B**), Labelled vM1 axons in the vibrissa primary somatosensory cortex (vS1). The pixel intensity of the axon signals was normalised to the peak value (green line). The signals of retrogradely labelled L2/3 and L5 neurons (arrowheads) were extracted from this measurement. Scale bar, 100 μm. (**C**), Laminar distribution of vM1 axons in vS1. Axons intensely innervated L1 and L5b, but sparsely innervated L4 compared to the mean of all layers (*P < 0.05, one sample t-test; 3 mice). (**D**), vM1 axons selectively innervate L1 and L5b of vS1. (**E**), vM1 infarction model made by the local irradiation of green light. Electrophysiological recordings from vS1 were performed at POD3 and POD14. *Right*, The infarction site was identified by cytochrome oxidase staining (1.4 mm anterior to the bregma, POD3). Scale bar, 1 mm; D, dorsal; L, lateral. (**F**), The means of the largest areas of infarction were 1.52 ± 0.26 mm^2^ at POD3 and 0.74 ± 0.08 mm^2^ at POD14 (P = 0.02, two sample t-test). Error bars are defined as SEM.

The projection pattern from vM1 to vS1 is not uniform but varies between layers in vS1^9,21^. To validate the layer dependency of the projection pattern from the vM1 infarction area, we injected an anterograde tracer, Biotinylated dextran amine (BDA), into vM1 (Fig. 1A) and identified vM1 axonal innervations in vS1. The signal intensity (green line in Fig. 1B) of the anterogradely labelled axons from vM1 was calculated. For this calculation, the signal of the soma, which is the result of a small amount of retrograde labelling by BDA^22^, was excluded. At the population level, the signal intensity was relatively stronger in L1 and L5b, but weaker in L4 compared to the mean intensity of all layers (N = 3) (Figs. 1C and S1). As summarised in Fig. 1D, vM1 axons projected densely to L1 and L5b.

### A photothrombotic infarction model in vM1

We made vM1 infarctions by using the photothrombotic method and recorded multi-unit activities (MUA) from vS1 at postoperative day 3 (POD3) and postoperative day 14 (POD14) (Fig. 1E). The sizes of the infarction areas centred at vM1 were stable on each experimental day but were significantly smaller at POD14 than at POD3. The means of the largest normalized areas were 1.52 ± 0.26 mm^2^ at POD3 (N = 5) and 0.74 ± 0.08 mm^2^ at POD14 (N = 4, P = 0.02, two-sample t-test, Fig. 1F). This reduction was also observed in other studies using photothrombotic infarctions^23,24^. Because we made the infarction to cover all vibrissae-related areas, the infarction area might have spread over to the other areas of vM1 such as forelimb or hindlimb. However, we did not observe any deficits of movements in these limbs.

### vM1 infarction disturbed temporal coding in vS1

**Figure 2:**
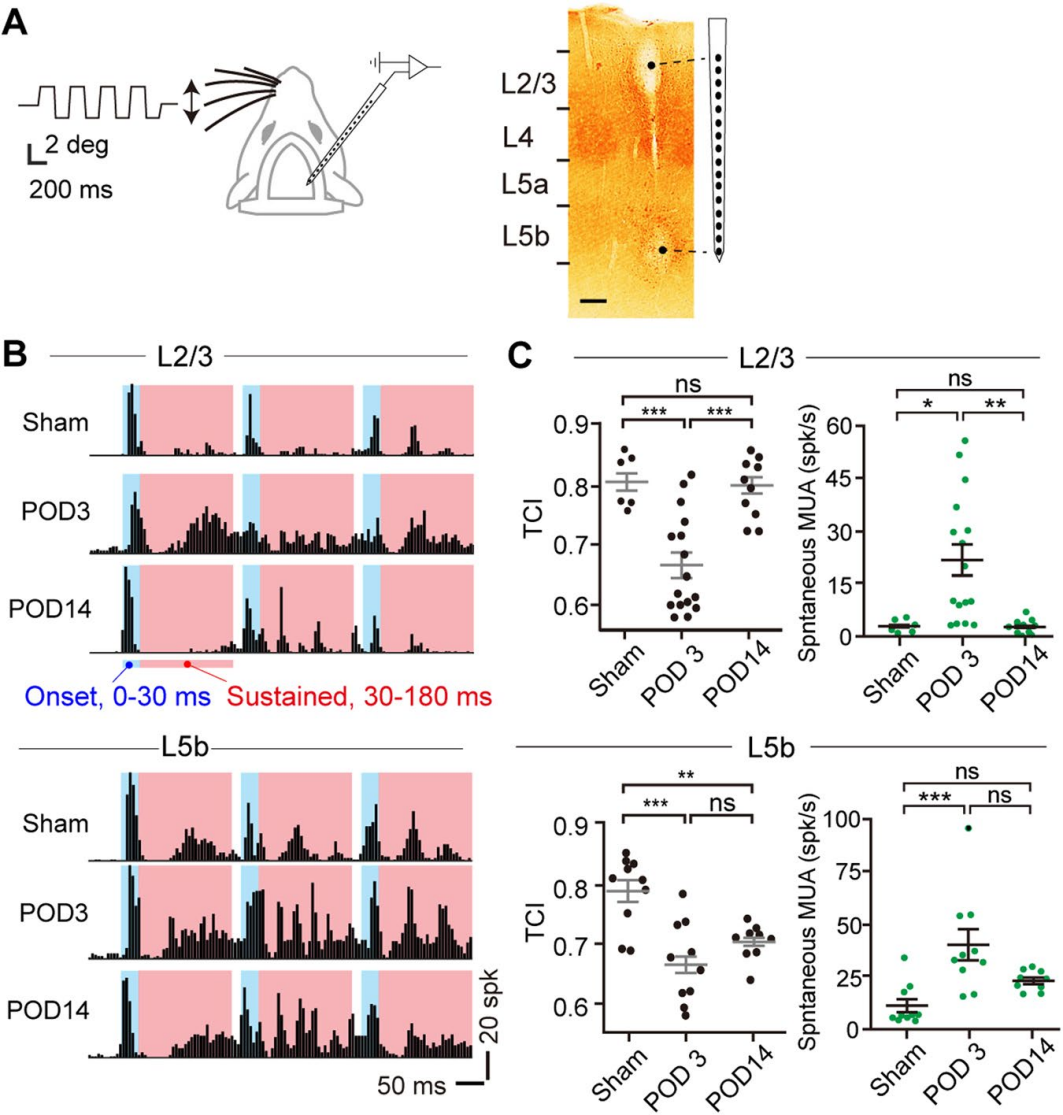
vM1 infarction disturbed temporal coding in vS1. (**A**), The whisker stimulation and 16-channel extracellular recording setup. *Left*, Directions of the whisker deflection (double-edged arrow) and a trace of the whisker position. *Right*, Electrolytic lesions at both ends of the recording sites. Scale bar, 100 μm. (**B**), Examples of peristimulus time histograms (PSTHs) of multiunit activity (MUA) evoked by whisker deflections in L2/3 (upper) and L5b (lower) from sham, POD3, and POD14 mice. MUA was classified into onset (blue area, 0–30 ms after the deflection onset) and sustained (red area, 30–180 ms after the deflection onset) responses. 5 ms/bin. **C**, Temporal coding index (TCI, left, black dots; see Methods) and spontaneous MUA (right, green dots) in L2/3 (upper) and L5b (lower) from sham (3 mice), POD3 (5 mice) and POD14 (4 mice). ***P < 0.001; **P < 0.01; Tukey’s honestly significant difference test. ns, not significant.

To measure the sensory processing of vibrissa inputs, MUA evoked by whisker deflections was recorded at different vS1 depths (Fig. 2A). In cortical L2/3 and L5b of sham mice, MUA was precisely time-locked to repetitive whisker stimuli (Fig. 2B). The time-course of the peristimulus time histograms (PSTHs) accurately reported the onset of vS1 inputs. In the acute phase of the vM1 infarction, for which MUA at POD3 increased with the onset response (0–30 ms after the onset of the stimulus) but less so than with the sustained response (30–180 ms after the onset of the stimulus) (Supplemental Table S1 and Fig. S2). Therefore, the onset of the next vibrissa inputs were relatively hard to identify from the PSTHs in either L2/3 or L5b (Fig. 2B). These changes in MUA at POD3 lowered the temporal coding index (TCI) (see Methods for the definition of TCI) (Fig. 2C). In the subacute phase of the infarction, which corresponds to POD14, the TCI was recovered to the sham level in L2/3 but not in L5b (Fig. 2C). In fact, among the observed regions, only in L5b did recovery not occur at POD14 (Table S1). These data indicate layer dependency in the M1 infarction effect. Noting that vM1 axons projected densely to L1 and L5b (Fig. 1D), our observations suggested that the loss of the synaptic inputs from vM1 to vS1 has an essential role in impairing sensory processing after vM1 infarction. Furthermore, spontaneous activities increased at POD3 but recovered to the sham level by POD14 (Fig. 2C). However, the recovery of spontaneous activity at POD14 in L5b was weak compared with other layers, which is consistent with the TCI, suggesting similar mechanisms might regulate TCI in different layers.

### Simulation of vS1 synaptic inputs from vM1

**Figure 3:**
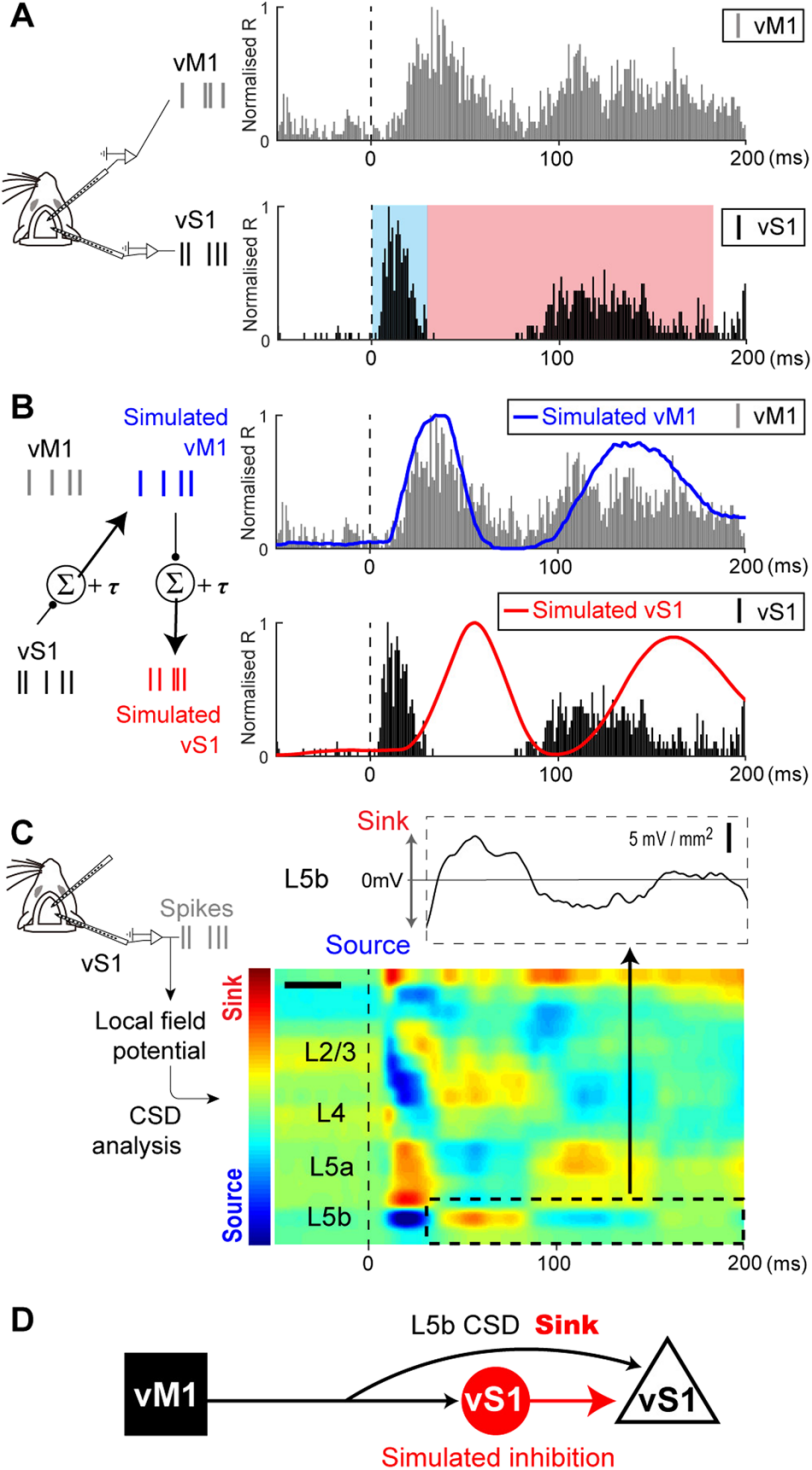
Simulation and CSD analysis indicate inhibition from vM1 to vS1. (**A**), Sensory-evoked responses simultaneously recorded from both vS1 and vM1. Recorded vM1 responses (upper, grey bars) and recorded vS1 L5b responses (lower, black bars) to whisker deflections (onset was set to zero). (**B**), *Left*, A schematic diagram for the simulation of vM1 and vS1 responses using an integrate-and-fire model from recorded vS1 responses. The simulated vM1 responses (blue line) positively correlated with the recorded vM1 responses (grey, the same as in A) (*r* = 0.68, P < 0.001). The simulated S1 responses (red line) negatively correlated with the recorded vS1 responses (black bars, the same as in A) (*r* = −0.52, P < 0.001). (**C**), The excitatory synaptic inputs in vS1 were revealed by CSD analysis as current sinks (red in the colourmap). Note, L5b of the CSD (dotted area in the colourmap and black line in the voltage graph) positively correlated with the simulated S1 responses in B (red line, *r* = 0.67, P < 0.001). (**D**), A schematic diagram of a network model from the simulation and CSD analysis. The excitatory synaptic input to pyramidal neurons in vS1 (black arrow) was observed as a current sink in the CSD. The input from inhibitory interneurons in vS1 was the simulated vS1 response (red arrow).

To determine the effect of vM1 on the sustained response in vS1, we first recorded MUA from vM1 and vS1 simultaneously in a sham animal (Fig. 3A,B). A sensory-evoked vM1 response (Fig. 3A, upper) was observed after the response in vS1 (Fig. 3A, lower) with a constant time delay. Furthermore, each onset and the sustained response was longer in vM1 than in vS1. These observations raised the possibility that vM1 receives excitatory inputs from vS1 with a constant delay and integrates them at a constant time^16,17^. To elucidate this possibility, we simulated the sensory-evoked vM1 responses from the vS1 responses by using an integrate-and-fire model based on excitatory synaptic connections in the direct pathway from S1 to M1 (see Materials and Methods and Fig. 3B)^21,25^. To maximize the correlation coefficient (*r*) between the recorded and simulated vM1 responses, the time window for integration (TW) and the time delay in firing (τ) were estimated to be 30 ms and 6 ms, respectively (Fig. S3). Using these values, the simulated vM1 responses (Fig. 3B, blue line) successfully reproduced the vM1 responses (Fig. 3B, grey bars, *r* = 0.68, P < 0.001). Therefore, it is likely that vM1 received sensory inputs directly from vS1, though we cannot rule out the possibility of the trans-thalamic pathway, in which S1 activates M1 through the posterior medial thalamic nucleus (POm)^26^.

We next conducted an additional simulation to predict vS1 responses from the simulated vM1 responses based on the excitatory synaptic connections from vM1 to vS1^21^. For this simulation, we again set TW = 30 ms and τ = 6 ms in the integrate-and-fire model based on excitatory synaptic connections from vM1 to vS1. If vS1 receives excitatory synaptic input, vS1 activity would increase according to the strength of the simulated synaptic input from vM1 (Fig. 3B, red line). However, unlike the vM1 responses, the recorded and simulated vS1 responses were negatively correlated (Fig. 3B, black bars and red line, respectively, *r* = −0.52, P < 0.001). In other words, the recorded vS1 response is suppressed with increasing simulated vS1 response. Thus, although there are both excitatory and inhibitory inputs from vM1 to vS1^14,19^, our results suggest that the net effect from vM1 on vS1 is relatively inhibitory.

To validate the credibility of this simulation, we performed current source density (CSD) analysis from the local field potentials recorded from vS1 to confirm if synaptic inputs from vM1 to vS1 exist. From the CSD analysis, we found excitatory inputs patterns to pyramidal neurons, which are considered the most likely generator of the CSD profile (see Methods)^27^. As such, CSD analysis can reveal excitatory synaptic inputs from vM1 as current sinks. The most promising candidate for the excitatory inputs from vM1 was vS1 L5b, where vM1 is innervated densely (Fig. 1C). The time course of the L5b CSD profile (dotted area in Fig. 3C) and that of the simulated S1 responses (red line in Fig. 3B) were positively correlated (*r* = 0.67, P < 0.001). This result indicates the validity of the vS1 simulation from vM1. In sum, it is likely that the net effect of inputs from vM1 is inhibitory via inhibitory interneurons (Fig. 3D)^14,28^.

### Inhibition from vM1 after infarction recovered temporal coding in vS1

**Figure 4:**
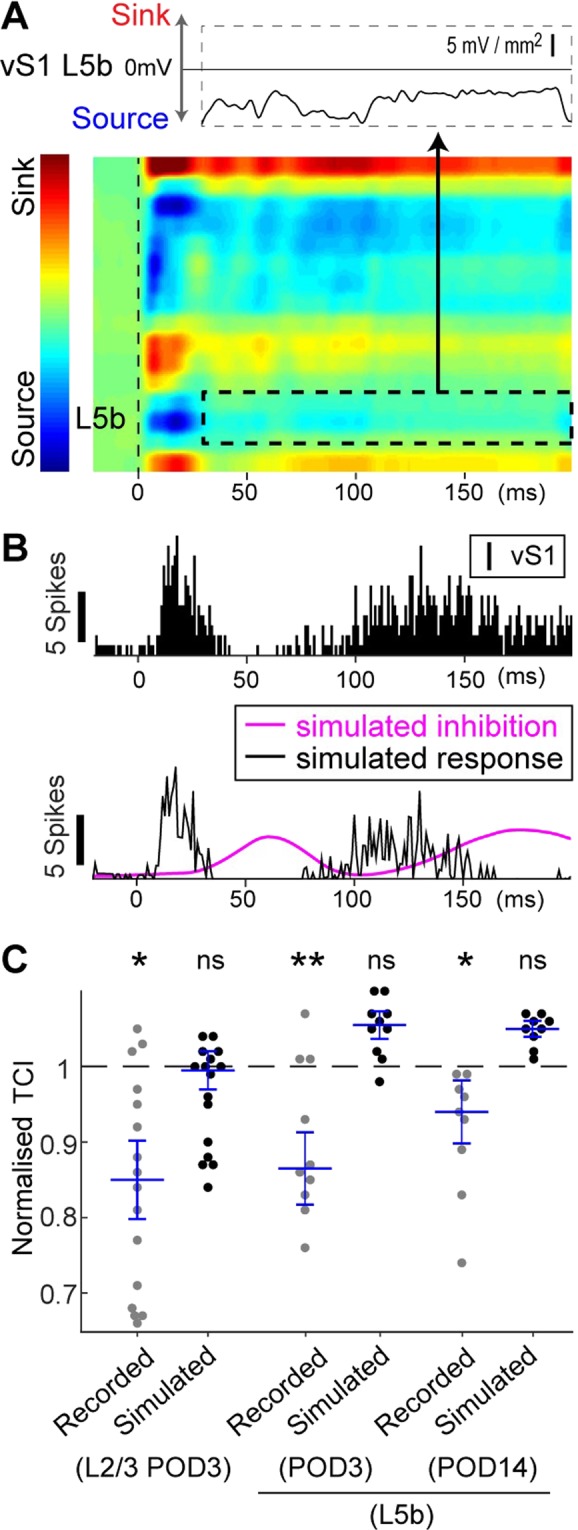
Application of inhibitory input to vM1 infarction can mimic temporal coding in sham animals. (**A**), The loss of excitatory inputs to vS1 L5b (dotted area) was visualised by CSD analysis at POD3. (**B**), *Upper*, An example of the recorded vS1 L2/3 responses at POD3. *Lower*, Simulated inhibition from vM1 (cyan line) suppressed the sustained responses. (**C**), TCI was recovered to the sham level in simulated vS1 responses: in L2/3, TCI at POD3 of recorded (0.85 ± 0.03) and simulated (1.00 ± 0.03); in L5b, TCI at POD3 of recorded (0.87 ± 0.03) and simulated (1.06 ± 0.01); in L5b, TCI at POD14 of recorded (0.94 ± 0.03) and simulated (1.05 ± 0.01). Each value was normalised to the level of sham mice. *P < 0.05; **P < 0.01; Dunnett’s test was used to compare with the sham level. ns, not significant. To calculate normalised TCI, the response to the first stimulus (from the stimulus onset until 180 ms) was used.

The results from the simulated vS1 response (Fig. 3) suggest the inhibition from vM1 to vS1 disappears in the case of vM1 infarctions. To confirm this hypothesis, we tested whether the simulated vS1 response could rescue the temporal coding if virtual inhibition from the simulated vM1 response was applied under the vM1 infarction condition. We first confirmed the loss of synaptic inputs to excitatory pyramidal neurons from vM1 by removing the current sink in L5b (Fig. 4A). Then, we simulated inhibition from vM1 to vS1 at POD3 (Fig. 4B). The result was a lowered sustained response and recovered temporal coding. At the population level, the simulated inhibition from vM1 effectively improved temporal coding to the sham level in L2/3 at POD3 and L5b at POD3 and POD14 (Fig. 4C). These results strongly suggest that the loss of inhibition from vM1 causes deficits in temporal coding in vS1.

### Verification of inhibition from vM1 using optogenetic activation

**Figure 5:**
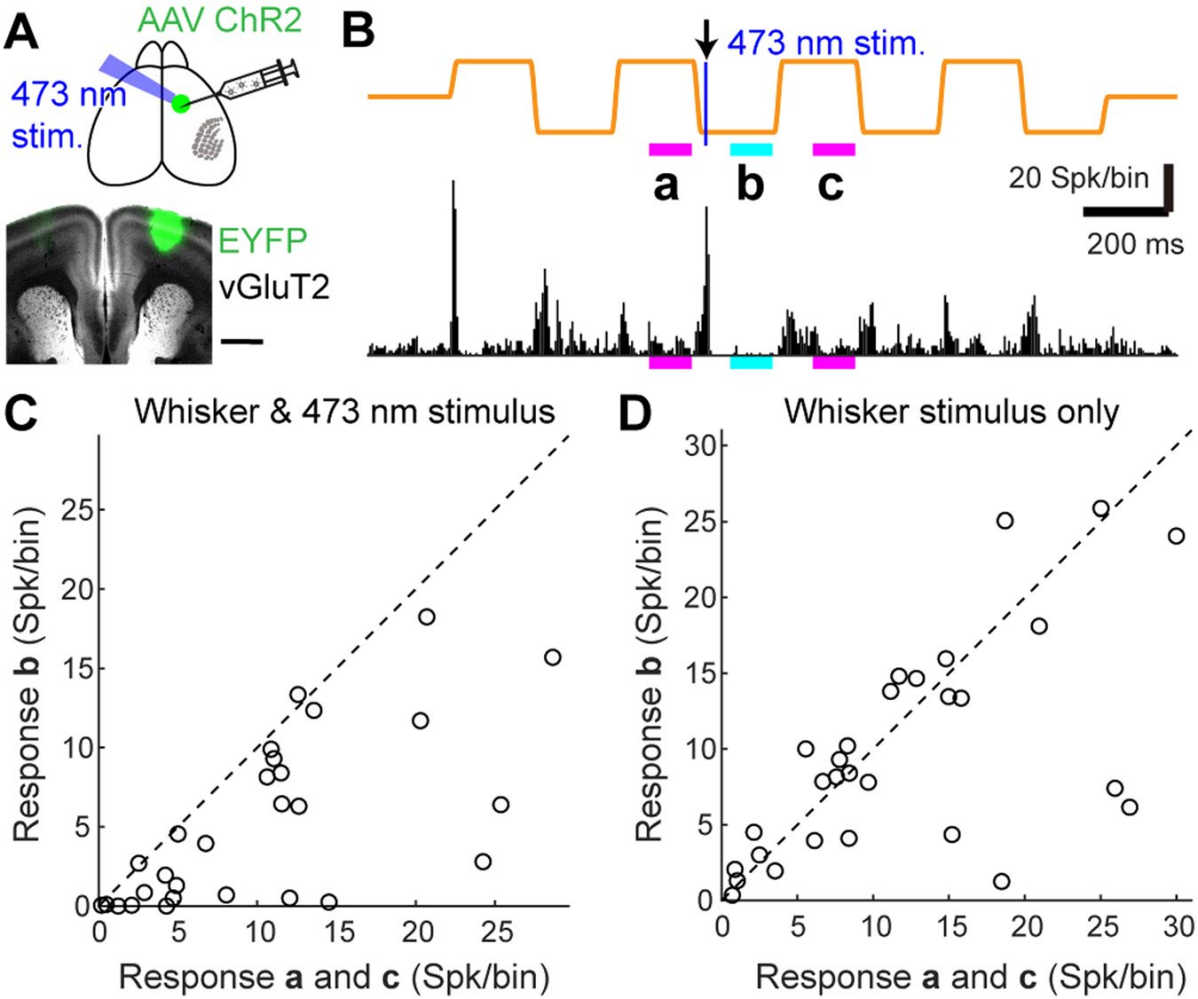
Optogenetic vM1 activation suppresses the sustained response to whisker stimulus in vS1. (**A**), Upper, schematic of the optogenetic activation. ChR2 was expressed in vM1 by virus-vector injection. The neurons in vM1 were activated by 473 nm light stimulus. Lower, an example of ChR2 expression in vM1 based on ChR2-EYFP (green) and vGluT2 (grey) staining. Scale bar, 1 mm. (**B**), An example of MUA in vS1 in response to whisker stimulus (piezo trace, orange) and 473 nm ChR2 activation in vM1 for 5 ms. The sustained responses at points (a–c) were used in the graphs seen in C and D. (**C**), The sustained responses at (b) (shown in **B**) were significantly suppressed compared with those at (a,c) (P < 0.001, N = 28 from four mice; Wilcoxon signed-rank test). (**D**), The sustained responses at (b) without ChR2 activation were not different from those at (a,c) (P = 0.48, N = 29 from four mice; Wilcoxon signed-rank test). The MUA of zero spikes at (a–c) was excluded from the analysis (N = 4 from C, N = 3 from D).

Our simulation studies suggest that vM1 suppresses the vS1 sustained response to a whisker stimulus. To verify the effect of vM1, we optogenetically activated vM1 neurons using virally expressing channelrhodopsin-2 (ChR2) in vM1 and observed the vS1 sustained response to a whisker stimulus (Fig. 5). The activation of vM1 suppressed the sustained response (b in Fig. 5B) compared with the prior- and post-sustained response without optogenetic activation (a and c in Fig. 5B). The suppression of the sustained response by 5 ms ChR2 activation in vM1 was statistically significant at the population level (P < 0.001, Fig. 5C). On the other hand, sustained responses without optogenetic activation (no light stimulation) were unchanged (P = 0.48, Fig. 5D). Moreover, a suppressive effect after optogenetic activation could be observed by optogenetic activation only (Fig. S4A,B). In mice without ChR2 expression, the light stimulation did not have any suppressive effects on the sustained responses (P = 0.50, N = 48 from three mice; Wilcoxon signed-rank test). Therefore, optogenetic activation of vM1 neurons intelligibly suppressed the sustained response in vS1 neurons.

## Discussion

In this study, we showed that the temporal coding of whisker-mediated sensory inputs in vS1 was impaired in the acute phase of vM1 infarctions and recovered in L2/3 to L5a but not in L5b in the subacute phase. A tracer study indicated that L5b in vS1 received dense innervation from vM1, while a simulation study and CSD analysis strongly suggested that vM1 infarction impairs temporal coding in vS1 by the loss of inhibition from vM1 to vS1. The effect of a reduced sustained response was confirmed by optogenetically activating vM1. These results show how stroke impairs the function of connected cortical areas and suggest the effective usage of sensory electrical stimulation (SES) in the acute phase of stroke for recovery^29,30^.

Motor infarctions occasionally cause sensory deficits. In the present study, we found that vM1 infarction increased the sustained response to whisker deflections more significantly than the onset response. This unbalanced increase between the two responses resulted in an impairment in temporal coding (Fig. 2). It has been reported that focal infarction of the neocortex induces disinhibition by widespread alternations in GABA_A_ receptor subtypes at various brain regions^31^, an effect that may underlie the reorganization of the somatotopy map in S1^4,5^. Meanwhile, vM1 directly modulates vS1 activity via disynaptic inhibition in normal animals^14,19^. Thus, these inhibitory mechanisms may be involved in the impairment of temporal cording upon vM1 infarctions. Especially, the disynaptic inhibition mechanism from vM1 may have a significant impact on temporal coding impairment because the inhibition from simulated vM1 could recover TCI in simulated vS1 responses under vM1 infarctions (Fig. 5). The physiological function of the sustained response in S1 is considered to be responsible for conscious sensory perception^32^. Moreover, it is thought to be a rebound response resulting from the recurrent activation of cortical and subcortical circuitry^33^ and controlled by connected areas^20^, such as inputs from vM1^15,34^. These studies support our result that vM1 infarctions largely influence the sustained responses in vS1.

Although a previous slice patch clamp recording study indicated both excitatory and inhibitory effects from M1 to S1^14^, most *in vivo* physiological studies have focused on the excitatory effect^15,35^, except the study simulating both effects by Zagha *et al*. (2016)^19^. We also observed an excitatory effect in vS1 by transient vM1 activation (Figs. 5B and S5A). However, the sustained responses were significantly reduced (Fig. 5). These results support the finding from our simulation study (Fig. 3) and the conclusion that inhibition from vM1 could help maintain temporal coding in vS1.

The recovery of temporal coding after vM1 infarction was similar to the data from a recent clinical study showing that sensory deficits observed in the acute phase of stroke largely recover over time^36^. However, the recovery process of our data was not uniform among layers. In the subacute phase, TCI in L2/3 recovered to the sham level, but not in L5b (Fig. 2). What is responsible for the difference in the recovery process among layers? A number of studies have shown that neural repair after stroke depends on tissue adjacent to or connected with the infarct lesion^37^. vS1 receives inputs not only from vM1 but also from the secondary somatosensory cortex (S2) and POm. vM1, S2, and POm have axons that ramify in L1 of vS1 and overlap with the apical dendrites of L2/3 and L5 pyramidal neurons^12,18,34,38^. Thus, we speculate that direct inputs from S2 or POm in L1 could compensate for the effect of M1 infarctions. Additionally, vM1 axons reside mostly in deep L5b and L6, whereas S2 axons comparatively project to L5a strongly, and L6 and POm axons are concentrated in L5a^14,17,39–41^ These observations support our CSD analysis data, which showed a reduction of excitatory inputs in L5b after infarction, suggesting a loss of inputs from vM1 (Fig. 4A). Although the apical dendrite of L5 neurons arbors within L2/3 and remodels its synapses^42^, it might be hard to compensate the loss of inputs to L5b after M1 infarction from other areas. Note, however, that we could not exclude the possibility that L5b neurons were affected by the plasticity of inputs from L1 after vM1 infarction. Thus, it is possible that inputs into L1 and L5b from vM1 neurons contributed to our observations. As such, we propose that layer-specific connectivity affects the recovery process after M1 infarction.

Sensory inputs are necessary for the successful execution and acquisition of skillful voluntary movements^3^. Therefore, re-establishing sensory processing and sensorimotor interactions in the infarction-damaged motor system appears to be essential for improving motor function. There is evidence indicating that SES improves motor function after infarction^2^. However, the optimal timing and protocol of SES are still debated. Our results may help explain why SES at 10–30 Hz increases corticospinal excitability^29^. As shown in Fig. S5, it takes about 42 ms after stimulation-evoked S1 responses to receive inhibition from M1 (21 ms from S1 to M1 and 21 ms from M1 to S1). This time corresponds to 24 Hz (1 / 0.042 s). Considering 24 Hz from the viewpoint of S1 inhibition from M1, SES at 30 Hz may be close to the threshold that effectively activates the cortico-cortical reciprocal circuit between M1 and S1. On the contrary, SES greater than 30 Hz does not excite M1-S1 circuits effectively, suggesting why 100 Hz SES is less effective than 10–30 Hz SES at exciting corticospinal neurons^29^. The results here may provide insight for an effective protocol of SES after stroke.

## Methods

### Animals

Male C57BL/6 mice (Sankyo Lab. Service Corp., Tokyo, Japan) 8–12 weeks old were used. All surgical procedures and postoperative care were performed following the guidelines of the Animal Care and Use Committee of Tokyo Women’s Medical University. Animal experiments were approved under the number AE17–127. All experiments were performed in accordance with relevant guidelines and regulations of the Animal Experiment Ethics Committee of Tokyo Women’s Medical University. Every effort was made to minimize the number and suffering of animals used in this study. The animals were housed in a room maintained at 23 ± 1 °C with a 12 h light/dark cycle. Food and water were available *ad libitum*.

### Anterograde labelling from vM1 to vS1

Biotinylated dextran amine (BDA; molecular weight 10,000; 10% in saline; Thermo Fisher Scientific, Waltham, MA, USA) was injected into the vM1 (1.4 mm anterior to the bregma and 1.1 mm lateral to the midline^9,21^) of normal animals. After a survival period of seven days, the mice were deeply anesthetized with sodium pentobarbital (60 mg/kg, intraperitoneally) and transcardially perfused by a fixative solution (4% paraformaldehyde and 0.2% picric acid in 0.1 M phosphate buffer). The brains were cut coronally into 40-μm sections. Sections were incubated overnight with a guinea pig monoclonal antibody against vesicular glutamate transporter type 2 (VGluT2) (1:500; VGluT2-GP-Af810; Frontier Institute Co., ltd., Ishikari, Japan) followed by Alexa Fluor 594-conjugated secondary antibody (for VGluT2; 1:500; Jackson ImmunoResearch, West Grove, PA, USA) and Alexa Fluor 488-conjugated streptavidin (Thermo Fisher Scientific), and subsequently with NeuroTrace 435/455 (1:100; Thermo Fisher Scientific). Cortical layer structures in vS1 were identified using cytoarchitecture, and the VGluT2 staining pattern was similar to that previously reported (see Fig. S2)^42^. Three serial sections were used to evaluate the pixel intensity of anterogradely labelled axons along the centreline of the barrel structure of vS1. The pixel intensity in each section was averaged and standardized with maximum values (three mice). These values were further normalized to the mean value of all layers.

### Photothrombotic infarction in mouse vM1

Each animal was anesthetized with an intraperitoneal injection of ketamine (100 mg/kg) and xylazine (16 mg/kg) and held in a stereotaxic apparatus. The skull was exposed and kept wet with saline on the surface to increase the transparency of the skull. Five minutes after the intraperitoneal injection of 1% rose bengal (100 mg/kg; Wako, Tokyo, Japan), green light coupled with an optic fibre (532 nm wavelength, 0.2 mm diameter, 4.5 mW; Thorlabs Inc., Newton, NJ, USA) was applied for 15 min to the right vM1 (1.4 mm anterior to the bregma and 1.1 mm lateral to the midline^9,21^) (Fig. 1)^23^. Subsequently, a head plate was glued onto the skull, and the animal was returned to the home cage. Infarct volumes were calculated using ImageJ software (https://imagej.nih.gov/ij/) by measuring the largest area of the infarct in all coronal sections, which were normalized by the ratio of ipsilesional to contralesional cortical volumes to exclude the shift effect of the cortex.

### *In vivo* electrophysiological recording

Electrophysiological recordings were performed at POD3 and POD14, which correspond to the acute and subacute phases of the infarction, respectively (Fig. 1). These times are analogous to the acute (<30 days, corresponding to the inpatient rehabilitation period) and subacute (60–90 days, corresponding to the typical outpatient therapy delivery) phases in human^6,30,43–45^. Each mouse was anesthetized with isoflurane (0.8–1.0% during recording) supplemented with an intraperitoneal injection of chlorprothixene hydrochloride (2 mg/kg) for sedation. The respiration rate was monitored and maintained at 80–110 breaths per minute by using a custom-made respiration monitor, 30 Hz USB camera and an acceleration monitor to detect the rib cage motion of the animal. The animals were maintained at 37 °C rectal temperature by a feedback-controlled heating pad. A silicone probe with 16 recording sites spaced 50 μm apart (A1 × 16–5mm-50–703; NeuroNexus, Ann Arbor, MI, USA) was inserted into vS1 (1.7 mm posterior to the bregma and 3.5 mm lateral to the midline, 35 degrees inclined laterally) until the tip reached a 900 μm depth from the pial surface. Then, local field potentials and MUA were obtained simultaneously from different cortical depths. To maintain the depth of anaesthesia during a recording, the respiration rate was controlled at 91 ± 9.3 (cycles/minute, mean ± S.D.) by changing the isoflurane concentration. In addition, to minimize the effect of a change in depth of anaesthesia, a recording session composed of 20 stimuli sequences was run twice, and the mean MUA was calculated between sessions. One session took over three minutes including the inter-stimulus interval. Therefore, the data are an average of at least six minutes.

### Spike detection

Data were recorded using a multichannel acquisition processor (Plexon Inc., Dallas, TX, USA). Local field potentials and multi-unit activity were separated using band-pass filtering at 0.5–300 Hz and 300–6000 Hz, and sampled at 1 kHz and 40 kHz, respectively.

For spike detection, a threshold was determined by the following equation:^46^

1 $${\rm{T}}{\rm{h}}{\rm{r}}{\rm{e}}{\rm{s}}{\rm{h}}{\rm{o}}{\rm{l}}{\rm{d}}=5\ast {\rm{m}}{\rm{e}}{\rm{d}}{\rm{i}}{\rm{a}}{\rm{n}}\,(|\,x\,|/0.6745)$$

Where x is the bandpass filtered signal. The spike number as multi-unit activity was counted and used for making peri-stimulus time histograms (PSTHs). In one animal, both M1 and S1 responses were recorded simultaneously by using two silicone probes (A1x16–5mm-50-703) for checking the validity of the simulation.

### Whisker stimulation

Whisker stimulations were generated using a piezoelectric device controlled by a custom-written MATLAB program (MathWorks, Natick, MA, USA). All whiskers were deflected forward and backward with a 170-ms steady-state after each deflection. This routine was cycled four times in one session, and the session was repeated 40 times at 3.6-s intervals. To evaluate the selectivity to deflection onset, the temporal coding index (TCI) was calculated as follows:

2 $${\rm{TCI}}={\rm{onset}}\,{\rm{response}}/({\rm{onset}}+{\rm{sustained}}\,{\rm{responses}}),$$

where the onset and sustained responses were the spike numbers during 0–30 ms and 30–180 ms after the whisker deflection, respectively. The responses to every deflection were used to calculate TCI. A TCI value close to one indicates that the MUA is time-locked to the onset of the deflection.

### Histological identification of laminar positions of recording sites

At the end of a recording, electrolytic lesions at both ends of the recording sites were made by delivering a small positive current (3 μA, 10 sec) to determine the exact laminar localization of the recording sites^47^. After the recording, the mouse was deeply anesthetized with sodium pentobarbital (60 mg/kg, intraperitoneally) and transcardially perfused by a fixative solution (4% paraformaldehyde and 0.2% picric acid in 0.1 M phosphate buffer). The brain was removed and post-fixed overnight at 4 °C. The brain was cut into 40-μm coronal sections using a vibratome (Leica VT1200S; Leica Microsystems, GmbH, Wetzlar, Germany). Sections were incubated overnight at 4 °C or 2–3 h at 37 °C with 0.05% 3, 3′-diaminobenzidine, 0.03% cytochrome c oxidase, and 4% sucrose in 0.1 M phosphate buffer, mounted on glass slides, and coverslipped using Eukitt (ORSAtec GmbH, Bobingen, Germany). Cortical layers in S1 were identified as follows using cytochrome c oxidase activity: 1) the identification of L4, which has visible barrel structures and the highest signal intensity; 2) the identification of L2/3 and L5a, which have a lower signal than L4; and 3) identification of L5b, which has a higher signal than L5a and L6. The recording sites located at the border between each layer were excluded from the analysis.

### Image acquisition and identification of S1 layer structures

Images were detected by a Zeiss epifluorescence microscope (Axio Scope.A1, Carl Zeiss, Oberkochen, Germany) equipped with a cooled CCD camera (RS 6.1, Quantum Scientific Imaging, Inc., Poplarville, MS, USA), acquired using μManager (http://www.micro-manager.org) and ImageJ software (https://imagej.nih.gov/ij), and saved as TIFF files. The contrast and brightness of the images were modified using Adobe Photoshop software (Adobe Systems Inc., San Jose, CA, USA). Layer structures in S1 were identified using cytoarchitecture and VGluT2 staining following a previous report:^48^ L1 has few cell bodies; L2/3 has a weaker VGluT2-staining than L4; L4 has VGluT2-positive barrel structures; L5a has weaker VGluT2-staining than L4 or L5b; L6 has smaller cell bodies than L5.

### Simulation analysis of inputs from vM1

To evaluate the role of vM1 in modulating S1 responses^14,15,20^, we simulated the effect of M1 inputs in two steps. First, we determined the parameters that explain well the responses transferred from S1 to M1 and vice versa. For this step, vM1 responses were simulated from the vS1 responses and compared with the recorded vM1 responses. Second, the effect of vM1 was simulated from the simulated vM1 responses. In both steps, the simulated responses were calculated using the integrate-and-fire model^25^.

The simulated vM1 response (M1simR) was calculated as a summation of vS1 responses (S1R) from the following equation:

3 $$M1simR(t)=\frac{1}{TW}\mathop{\sum }\limits_{i=1}^{TW}S1R(t-i-\tau ),$$

where TW is the time window (ms) for integrating the presynaptic inputs, i.e., the summation of spike activity, and $\tau$ is the time delay to fire (ms) between vM1 and vS1 responses.

Inversely, the simulated S1 response derived from vM1 response (S1simR) was calculated as a summation of the vM1 responses. Because we assumed bidirectional connections between vM1 and vS1 in the simulation, we applied the same value to TW and $\tau$ derived fromM1simR. S1simR is defined as:

4 $$S1simR(t)=\frac{1}{TW}\mathop{\sum }\limits_{i=1}^{TW}M1simR(t-i-\tau ).$$

Although there is no direct evidence to apply the same TW and $\tau$ to the simulation from the vM1 inputs, validation of this decision was performed by estimating the synaptic inputs into vS1 shown below. It is difficult to predict spontaneous activity in vM1 from the vS1 response. This reason is that the driving force of the whisker response in vM1 mainly comes from vS1, whereas that of spontaneous activity comes not only vS1 but other basic circuits. Therefore, we could not include spontaneous activity into the simulation.

### Current source density analysis

Current source density (CSD) analysis was used to detect the time and the cortical layer of the synaptic inputs, which was observed as the current sink^27^. At a certain cortical depth (z), the relation between the estimated CSD, $\hat{C}(z)$, and measured potential, $\varnothing (z)$, can be estimated as follows:

5 $$\hat{C}(z)\approx -\frac{\varnothing (z+h)-2\varnothing (z)-\varnothing (z-h)}{{h}^{2}},$$

where ${\rm{h}}$ is the distance between adjacent recording sites (50 μm in this study). Recording trials were repeated 40 times in each experiment to obtain averaged CSD. We estimated the CSD at the top and bottom electrode contacts by the method of Vaknin *et al*.^49^. A three-point Hamming filter was used to decrease spatial noise^50^. Data are represented as a pseudo-colour code from red (sink) to blue (source).

### Optogenetic activation

The adeno-associated virus AAV-DJ-hSyn-hChR2(H134R)-EYFP was generated from the plasmid pAAV-hSyn-hChR2(H134R)-EYFP (Addgene Stock No. 26973) to transfect M1 neurons. C57BL/6 mice were anesthetized with a mixture of ketamine (100 mg/kg) and xylazine (16 mg/kg), and a small craniotomy was made in the right M1 (lateral 1.1 mm and anterior 1.4 mm from Bregma). Virus-containing solution was injected at two sites (50 nL each) of different depths (230 and 400 μm) from the brain surface with a stereotaxic injector (QSI; Stoelting, Wood Dale, IL, USA). The time of stimulation was controlled by the shutter (LS3, Vincent Associates) in free space. After three weeks of viral expression, animals were prepared for vS1 recording as described above. Light-activation of vM1 was achieved by an optic fibre placed on the skull. Fibre output (~35 mW) was checked using a power meter and controlled to keep the response level under that of whisker stimulus to prevent negative effects by the excessively strong activation of vM1 neurons (Fig. S4C). For combined whisker and light stimuli, the light stimus was delayed at 30 msec from the onset of the fourth piezo deflection to mimic the vM1 response and compare the sustained response before and after the light stimulus.

### Statistical methods

Data are represented as the mean ± standard error (SEM). Statistical analyses were performed using SPSS 25.0 (SPSS Inc., Chicago, IL, USA). The specific tests used are stated alongside all probability values reported. Differences from baseline values were analyzed using the one-sample t-test (Fig. 1C). Mean infarction area was compared by two-sample t-test (Fig. 1F). MUA and TCI were analyzed by one-way ANOVA followed by either Tukey’s honestly significant difference test (Figs. 2C, S1, Table S1) or Dunnett’s test (Fig. 4C) for multiple comparison. Correlation between recorded and simulated MUA was also calculated with Spearman’s rank correlation analysis (Fig. 4B). Comparisons between the responses with and without light stimulation were analysed by Wilcoxon-signed rank test (Fig. 5). A p value < 0.05 was considered significant.

## Supplementary information

Supplemental material.

## Data Availability

The datasets generated during and/or analyzed during the current study are available from the corresponding authors upon reasonable request.
