# Supplementary material for: Layer-specific sensory processing impairment in the primary somatosensory cortex after motor cortex infarction
Source: Sci Rep. 2020 Feb 28;10:3771. doi: 10.1038/s41598-020-60662-7 (PMC7048762; doi:10.1038/s41598-020-60662-7)
Supplement: Supplementary file 1 — Supplemental material. [file 41598_2020_60662_MOESM1_ESM.pdf]

SUPPLEMENTAL MATERIAL

Layer-specific sensory processing impairment in the primary somatosensory cortex after motor cortex infarction

Atsushi Fukui[1,2,3†]; Hironobu Osaki[3†*]; Yoshifumi Ueta[3]; Kenta Kobayashi[4,5]; Yoshihiro Muragaki[1,2]; Takakazu Kawamata[2]; Mariko Miyata[3*]

[1]Faculty of Advanced Techno-Surgery, Institute of Advanced Biomedical Engineering and Science; [2]Department of Neurosurgery; [3]Division of Neurophysiology, Department of Physiology, Graduate School of Medicine, Tokyo Women's Medical University, Tokyo 162-8666, Japan; [4]Section of Viral Vector Development, National Institute for Physiological Sciences, Okazaki 444-8585, Japan; [5]SOKENDAI (The Graduate University for Advanced Studies), Hayama 240-0193, Japan

Supplemental figures

Figure S1

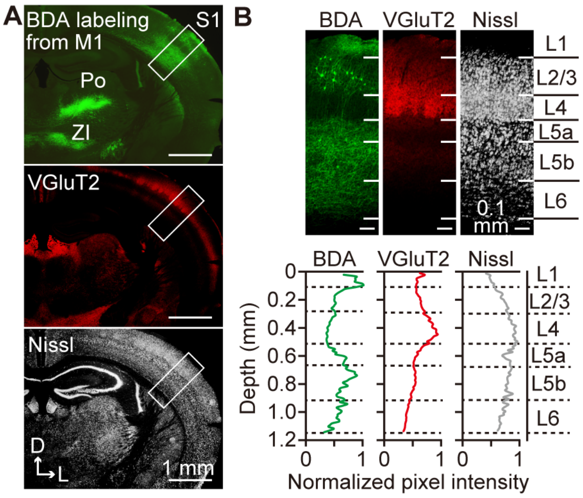

A, Anterogradely-labeled axons by BDA (biotinylated dextran amine) injection from vibrissa M1. Vibrissa S1 area and its layer structure were identified by VGluT2 and Nissl staining (B). Note that labelled axons in POm and zona incerta (ZI) indicate exact localisation of tracer injection in M1. **B**, Identification of S1 layers by VGluT2 and Nissl staining. The intensity of fluorescent signals for BDA, VGluT2, and Nissl across layers.

Figure S2

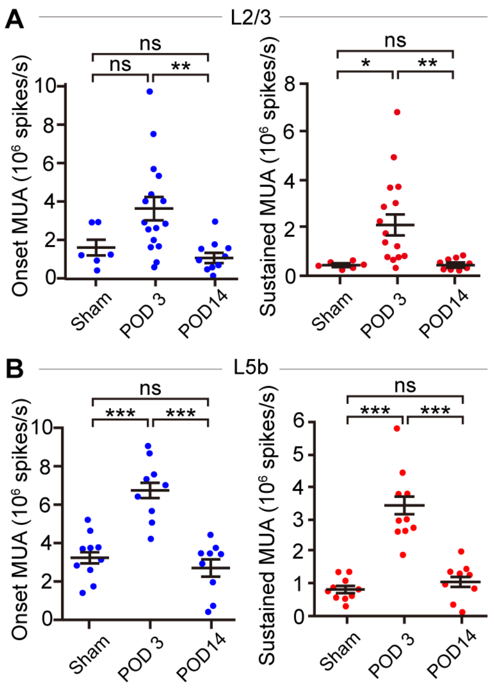

A, MUA evoked by whisker deflection at the onset (0–30 ms) and sustained (30–180 ms) in L2/3 of sham (6 recordings from 3 mice), POD3 (16 recordings from 5 mice) and POD14 (10 recordings from 4 mice). **B**, MUA in L5b of sham (10 recordings from 3 mice), POD3 (10 recordings from 4 mice) and POD14 (9 recordings from 5 mice). ***, $P < 0.001$; TTukey`s honestly significant difference test. ns, not significant.

Figure S3

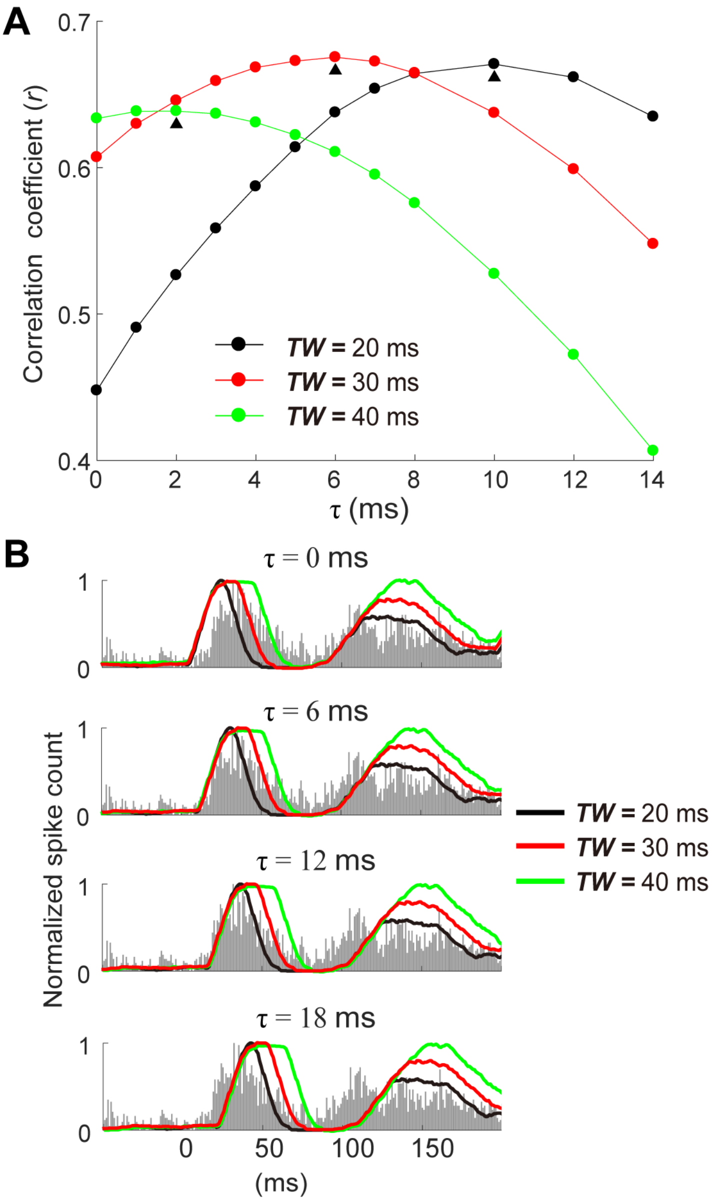

A, The values of the correlation coefficient (*r*) between recorded and simulated vM1 responses varied according to the parameters in the integrate-and-fire model. The *r* is highest at the integration time window (*TW*) was 30 ms the delay to fire (τ) was 6 ms. Triangles indicate peak points of the *r* in each *TW*. The *r* was calculated between 0 to 200ms from the stimulus onset. **B**, PSTHs for the simulated M1 responses at various *TW* and τ overlaid on PSTHs for the recorded ones (grey bars, 1 ms/bin).

Figure S4

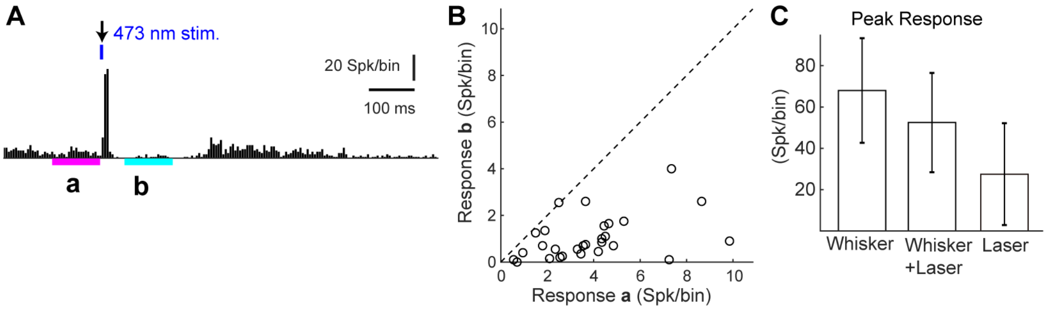

A, The vS1 MUA induced by the activation of vM1 optogenetically. **B**, The MUA at **b** (shown in panel **A**) was lower than that of **a**. **C**, The peak responses using the optogenetical laser stimulus were not higher than that of whisker stimulus.

Figure S5

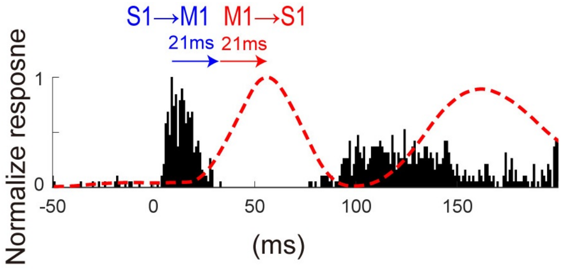

The simulated inhibitory effect from M1 (red dotted line) was delayed 42 ms (21+21ms) from recorded S1 responses (black PSTH).

Supplementary table

Table S1. Multiunit activity (MUA) recorded from different layers of mouse vS1

	MUA	sham	poststroke	poststroke	Statistical comparisons		
	(10^6spikes/s)	(a)	(b) POD3	(c) POD14	a vs. b	b vs. c	a vs. c
L2/3	Baseline MUA	2.75 ± 0.73	21.2 ± 4.42	2.56 ± 0.67	a < b	b > c	n.s.
		(n = 6)	(n = 16)	(n = 10)	(P = 0.014)	(P = 0.003)	(P = 0.999)
	Onset MUA	1.71 ± 0.43	3.76 ± 0.62	1.29 ± 0.26	n.s.	b > c	n.s.
		(n = 6)	(n = 16)	(n = 10)	(P = 0.078)	(P = 0.008)	(P = 0.900)
	Sustained MUA	0.34 ± 0.06	2.07 ± 0.43	0.31 ± 0.10	a < b	b > c	n.s.
		(n = 6)	(n = 16)	(n = 10)	(P = 0.018)	(P = 0.004)	(P = 0.999)
	Onset/total ratio	0.82 ± 0.02	0.67 ± 0.02	0.80 ± 0.02	a > b	b < c	n.s.
		(n = 6)	(n = 16)	(n = 10)	(P < 0.001)	(P < 0.001)	(P = 0.925)
L4	Baseline MUA	2.37 ± 0.78	28.01 ± 4.88	11.85 ± 2.51	a < b	b > c	n.s.
		(n = 8)	(n = 17)	(n = 12)	(P < 0.001)	(P = 0.017)	(P = 0.34)
	Onset MUA	3.95 ± 0.48	6.99 ± 0.53	4.00 ± 0.46	a < b	b > c	n.s.
		(n = 8)	(n = 17)	(n = 12)	(P = 0.002)	(P < 0.001)	(P = 0.998)
	Sustained MUA	0.99 ± 0.15	4.15 ± 0.53	1.53 ± 0.12	a < b	b > c	n.s.
		(n = 8)	(n = 17)	(n = 12)	(P < 0.001)	(P < 0.001)	(P = 0.717)
	Onset/total ratio	0.80 ± 0.01	0.65 ± 0.02	0.71 ± 0.02	a > b	b < c	a > c
		(n = 8)	(n = 17)	(n = 12)	(P < 0.001)	(P=0.015)	(P = 0.006)
L5a	Baseline MUA	22.34 ± 5.51	56.75 ± 9.23	29.30 ± 3.04	a < b	b > c	n.s.
		(n = 6)	(n = 11)	(n = 8)	(P = 0.015)	(P = 0.036)	(P = 0.831)
	Onset MUA	3.61 ± 0.50	5.66 ± 0.44	3.19 ± 0.49	a < b	b > c	n.s.
		(n = 6)	(n = 11)	(n = 8)	(P = 0.022)	(P = 0.003)	(P = 0.837)
	Sustained MUA	1.34 ± 0.24	3.69 ± 0.39	1.46 ± 0.23	a < b	b > c	n.s.
		(n = 6)	(n = 11)	(n = 8)	(P < 0.001)	(P < 0.001)	(P = 0.969)
	Onset/total ratio	0.74 ± 0.02	0.61 ± 0.02	0.68 ± 0.01	a > b	b < c	n.s.
		(n = 6)	(n = 11)	(n = 8)	(P < 0.001)	(P = 0.011)	(P = 0.106)
L5b	Baseline MUA	10.54 ± 3.06	39.46± 7.36	22.51 ± 1.61	a < b	n.s.	n.s.
		(n = 10)	(n = 10)	(n = 9)	(P < 0.001)	(P = 0.054)	(P = 0.214)
	Onset MUA	3.30 ± 0.38	6.69± 0.49	2.73 ± 0.47	a < b	b > c	n.s.
		(n = 10)	(n = 10)	(n = 9)	(P < 0.001)	(P < 0.001)	(P = 0.662)

Sustained MUA	0.86 ± 0.11	3.38 ± 0.36	1.11 ± 0.19	a < b	b > c	n.s.
	(n = 10)	(n = 10)	(n = 9)	(p < 0.001)	(p < 0.001)	(P = 0.767)
Onset/total ratio	0.79 ± 0.02	0.67 ± 0.02	0.70 ± 0.01	a >b	n.s.	a > c
	(n = 10)	(n = 10)	(n = 9)	(P < 0.001)	(P = 0.242)	(P = 0.007)

Data are mean ± SEM. Baseline MUA, MUA before whisker stimulation; Onset MUA, MUA of <30 ms from whisker stimulation; sustained MUA, MUA of 30–180 ms from stimulation; total, summation of onset and sustained MUA. n, number of recorded channels from 3, 5 and four mice for sham, postoperation day 3 (POD3), and POD14, respectively. P value, Tukey–Kramer test. n.s., not significant.
